# Prevalence of Cardiovascular Disease and Associated Factors Among Type 2 Diabetes Patients in Selected Hospitals of Harari Region, Eastern Ethiopia

**DOI:** 10.3389/fpubh.2020.532719

**Published:** 2021-02-05

**Authors:** Lemma Demissie Regassa, Assefa Tola, Yohanes Ayele

**Affiliations:** ^1^Department of Epidemiology and Biostatistics, School of Public Health, College of Health and Medical Sciences, Haramaya University, Harar, Ethiopia; ^2^Department of Clinical Pharmacy, School of Pharmacy, College of Health and Medical Sciences, Haramaya University, Harar, Ethiopia

**Keywords:** type 2 diabetes mellitus, prevalence, cardiovascular diseases, Ethiopia, hospitals, Harar

## Abstract

**Background:** Cardiovascular disease (CVD) is the most prevalent complication and the leading cause of death among patients with diabetes mellitus (DM). Type 2 diabetes mellitus (T2DM) patients have a 2- to 4-fold increased risk of CVD. There is a scarcity of data about the magnitude of CVD among patients with diabetes in Ethiopia. This study aimed to assess the prevalence and associated factors of CVD among T2DM patients at selected hospitals of Harari regional state of Ethiopia.

**Methods:** This hospital-based retrospective data review was conducted among T2DM patients on follow-up in the diabetes clinics of selected hospitals of Harari regional state. The records of T2DM patients who have been diagnosed between January 1, 2013, and December 31, 2017, were reviewed from March to April 2018. Data were collected by using structured checklists from all necessary documents of T2DM patients. Statistical analysis was done using STATA 14.1. Bivariate and multivariate logistic regressions were used to identify factors associated with CVD.

**Result:** The records of 454 T2DM patients were extracted from three government hospitals in Harari regional state. Their age was ranging from 15 to 86 years with a mean age (±*SD*) of 45.39 (14.76). The overall prevalence of CVD among T2DM patients was 42.51%, composed of hypertensive heart diseases (38.99%), heart failure (6.83%), and stroke (2.20%). The final multivariate logistic regression model revealed that age older than 60 years [adjusted odds ratio (AOR) = 3.22; 95% CI: 1.71–6.09], being physically inactive (AOR = 1.45; 95 CI: 1.06–2.38), drinking alcohol (AOR = 2.39; 95% CI: 1.17–6.06), hypertension (AOR = 2.41; 95% CI: 1.52–3.83), body mass index >24.9 kg/m^2^ (AOR = 1.81; 95% CI: 1.07–3.07), and experiencing microvascular diabetic complications (AOR = 3.62; 95% CI: 2.01–6.53) were significantly associated with the odds of having CVD.

**Conclusion:** The prevalence of CVD was high and associated with advanced age, physical inactivity, drinking alcohol, higher body mass index, hypertension, and having microvascular complications. Health care workers should educate T2DM patients about healthy lifestyles like physical activity, weight reduction, blood pressure control, and alcohol secession, which can reduce the risk of CVD.

## Introduction

Type 2 diabetes mellitus (T2DM) is a progressive and chronic metabolic disorder that is characterized by insulin resistance and functional failure of pancreatic beta cells (1). The prevalence of T2DM has been increasing intensely over the past few decades, with the highest rates of growth being seen in Sub-Saharan Africa (2, 3).

Cardiovascular disease (CVD), which involves heart and blood vessels, includes coronary heart disease (CHD), cerebrovascular disease, peripheral arterial disease, deep vein thrombosis, and pulmonary embolism (4, 5). It is the main cause of complications and morbidity among patients with T2DM globally (6, 7). Among T2DM patients, CVD risk was estimated to be 2- to 4-fold higher than the non-diabetic population (8, 9).

The natural history of T2DM is a slow process and may last even a decade; it might be initially presented with macroangiopathy, particularly CHD (10–13). The effect of T2DM on cardiovascular manifestations varies based on the specific cardiovascular outcome. Atherosclerosis, the major cause of macrovasculature, is the result of metabolic syndrome in diabetes patients (14–16). Similarly, alterations of small vessels in the brain, heart, and peripheral vasculature are contributing to the development of CVD and mortality (15). Another reason for the occurrence of CVD is inflammation, as immune response occasionally resulted in a detrimental effect. Even though DM is characterized by low-level inflammation, there is evidence showing that the immune activation preceding insulin resistance in diabetic and pre-diabetic states increases cardiovascular risk in T2DM processes (17). In addition to the impact of DM, modifiable and non-modifiable factors are contributing to the causation of CVD (18, 19).

Although microvascular complications have a significant role in the prognosis of T2DM, CVDs are the leading cause of morbidity and mortality among patients living with T2DM (6, 20). More than 70% of hospitalizations for chronic complications of diabetes are attributable to CVD (21, 22). The risk of morbidity and mortality caused by CVD in diabetes patients increases with the long duration of the diabetes (23–25).

Even though epidemiological studies have demonstrated an association between CVD and blood glucose levels, studies that indicate the magnitude of CVD and associated factors among diabetes patients in Harari region are limited. Thus, we aimed to assess the prevalence and associated factors of CVD among T2DM patients in hospitals of Harari regional state of Ethiopia.

## Materials and Methods

### Study Area and Period

This study was conducted among T2DM patients on follow-up in the diabetes clinics of government hospitals of Harari regional state. Harari is the smallest of the nine states of Ethiopia located in the eastern part of the country and surrounded by the east Hararghe zone of Oromia regional state. In the region, there are two public hospitals, one federal police hospital, two private hospitals, and eight health centers. This study was conducted in two public hospitals and one federal police hospital, namely, Hiwot Fana Specialized University Hospital (HFSUH), Jugal General Hospital (JGH), and Federal Harar Police Hospital (FHPH).

HFSUH is the teaching hospital for Haramaya University and comprehensive hospital for East Ethiopia (including Harari region, some parts of Somali region, and eastern Hararghe zone of Oromia) that is expected to serve about 5.8 million people in the eastern part of Ethiopia (26). Similarly, JGH is the oldest hospital in the country. FHPH is the government hospital serving police communities and their families. Hospitals are serving diabetes patients under the established chronic follow-up clinics. Patients' records from January 2013 to December 2017 were extracted in March to April 2018.

### Study Design

Hospital-based retrospective data review was conducted on records of T2DM patients at government hospitals of Harari regional state of Ethiopia.

### Population and Selection Criteria

Records of T2DM patients who were diagnosed after January 1, 2013, and before December 31, 2017, were included, but patients with no baseline records were excluded. Additionally, patients with body mass index (BMI) <18.5 kg/m^2^, end-stage renal diseases, transplanted organs or on dialysis, and/or other diagnosed chronic diseases like human immunodeficiency virus (HIV)/acquired immunodeficiency syndrome (AIDS), chronic obstructive pulmonary disease (COPD), or chronic liver disease (cirrhosis) were also excluded, as these factors lead to the immune deficiency.

### Sampling Technique and Sample Size Determination

The sample size was calculated by Epi Info version 7 using single and double population proportion formula taking 95% confidence level, 80% power, and 5% precision. For the first objective, the calculated sample size was 376, which was based on the 42.6% prevalence of CVD in North India during 2011–2014 (27). For the second objective, the maximum sample was found for the obesity (≥30 kg/m^2^) (*N* = 334). By adding the 20% for incomplete records for CVD, the minimum sample size calculated was 454. Patients were selected by simple random sampling from the registry of the follow-up using computer-generated numbers. Patient identification number (medical record identifier) was randomized by using Excel to select an individual patient.

### Operational Definition

#### Cardiovascular Disease

CVD comprises the major disorders of the heart and the arterial circulation supplying the heart, brain, and peripheral tissues. Thus, CVD will be considered if the patient had at least one but not limited to hypertensive heart diseases, heart failure, or stroke (4, 28).

#### Physical Activity

classified based on the occupational status of the patients (29). Physical activities were merged and regrouped into three categories: both moderate and high physical activity are leveled as “physically active” and those whose score is within the range of sedentary life are termed as “physically inactive.”

#### Hypertension (HTN)

defined as systolic blood pressure >139 mmHg and/or diastolic blood pressure >89 mmHg.

#### Controlled Blood Pressure

defined as systolic blood pressure between 120 and 139 mmHg and diastolic blood pressure between 65 and 85 mmHg (30, 31).

#### Controlled Blood Glucose

Glycemic status was categorized as good glycemic control if average (3-months average) fasting blood glucose (FBG) is 80–130 mg/dl (4.4–7.2 mmol/L) and poor control if FBG was >130 mg/dl (>7.2 mmol/L) (32).

#### Body Mass Index

The BMI is reclassified as normal if BMI is between 18.5 and 24.9 kg/m^2^ (18.5–24.9 kg/m^2^) and above normal if BMI is above 24.9 kg/m^2^ (33).

### Data Collection Methods

The data were collected by using structured checklists from T2DM patients' documents including DM registration book, electronic information databases, patient card, and follow-up records. Data were collected by health officers and nurses working in the respective hospitals but not at diabetes clinics. All the filled extraction sheets were checked for completeness and consistency by supervisors and investigators to ensure the quality of data. We also cross-checked the data entry and clarified any missing data.

### Data Management and Analysis

**Figure 1:**
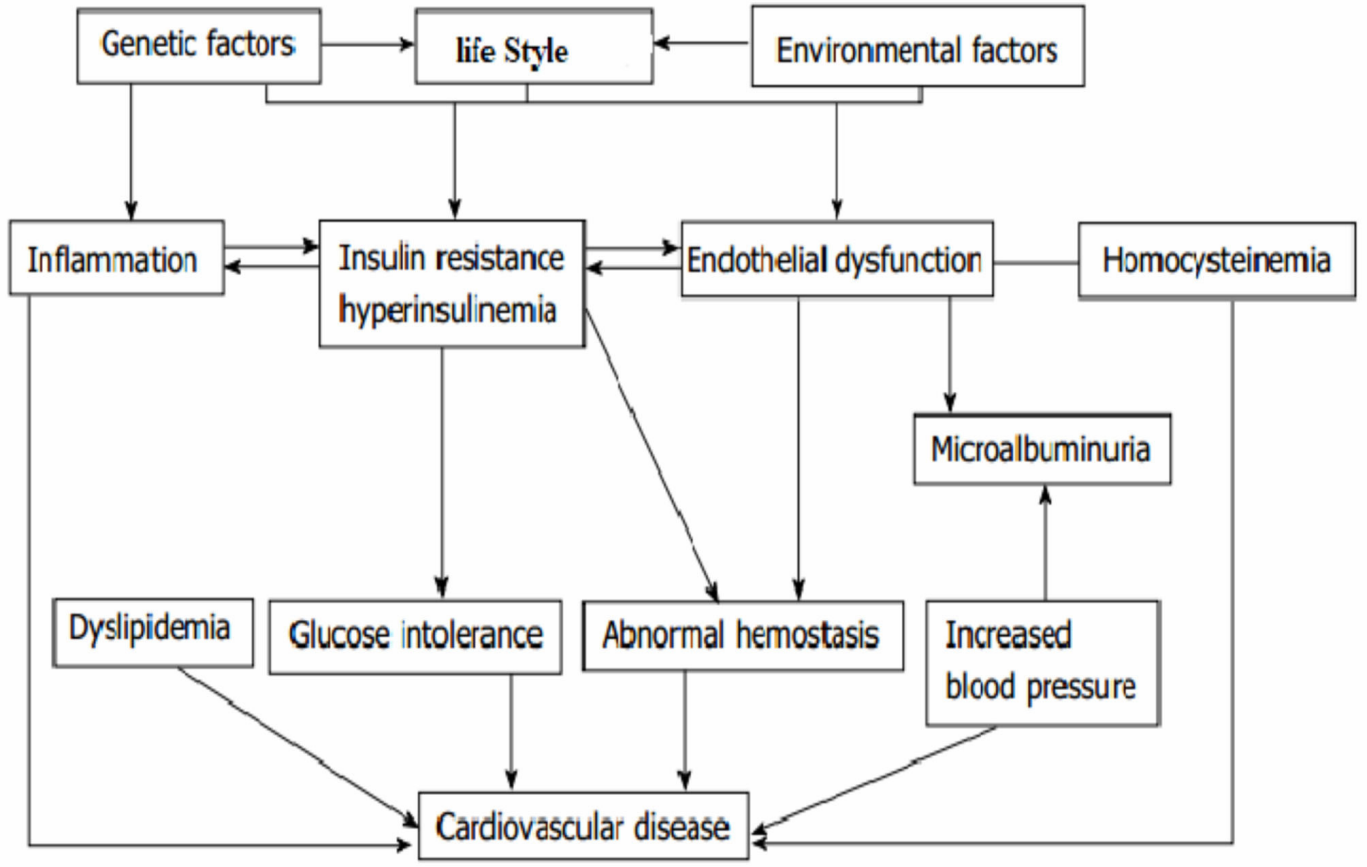
Conceptual framework for the causation and risk factors of cardiovascular disease in type 2 diabetes. Source [different kinds of literature (28, 34, 35)].

Statistical analysis was done using STATA 14.1. The risks of CVD and sociodemographic characteristics were summarized using proportion and mean with standard deviations. Outcome variable is determined if a patient experienced at least one type of CVD (coronary artery diseases, hypertensive heart diseases, stroke, heart failure, or any other else). To determine the factors for CVD, bivariate and multivariate logistic regressions were fitted, and variables were selected using the acyclic graph model selection (Figure 1). The final optimal model was selected based on Akaike information criterion (AIC) (36). Hosmer and Lemeshow test was fitted to test model fitness, and appropriate methods of multicollinearity test between independent variables were applied.

## Results

### Sociodemographic Characteristics

Total records of 454 T2DM patients were extracted from three government hospitals: 230 (50.66%) HFSUH, 111 (24.45%) JGH, and 113 (24.89%) FHPH. Two hundred fifty-nine (57%) were males. The age ranges from 15 to 86 years with mean (±*SD*) of 45.39 (14.76). The median age was 50 years (q1:30, q3:60). The majority (74.67%) of the patients where older than 40 years, and only 44 (9.69%) were younger than 30 years. The majority (73.84%) of the patients were urban dwellers and 389 (85.68%) were currently married. Regarding the occupation, 186 (40.97%) were civil servants, while 177 (39%) had private work (Table 1).

**Table 1:** Sociodemographic characteristics of type 2 diabetes patients in government hospitals of the Harari region, Eastern Ethiopia.

**Sociodemographic characteristics**	**Categories**	**Frequency**	**Percentage (%)**
Sex	Male	259	57.05
	Female	195	42.95
Age	Mean (±SD)	48.39 (14.76)	
Residence	Urban	333	73.84
	Rural	118	26.16
Marital status	Married	389	85.68
	Non-married^a^	65	14.32
Occupation	Civil servants	186	40.97
	Private workers^b^	177	38.99
	Unemployed	64	14.10
	Retired^c^	27	5.95

a Could be single, widowed, divorced, or separated.

b Are farmers, merchants, and other self-employments.

c *Retired individuals used to work in the government or non-governmental organization and terminates their job due to reaching the age of retirement*.

### Clinical Characteristics and Risks of Cardiovascular Complication

Most (93.17%) patients started their DM follow-up immediately after diagnosis, but 6.83% delayed from 1 month to a year. Metformin alone (38.55%) and metformin in combination with glibenclamide (49.56%) were the commonly utilized therapies. From the total, 115 (25.39%) had a family history of DM. Majority (68.81%) of the patients' FBS level was above 130 mg/dl, while only 128 patients (28.19%) have a controlled FBS level.

Regarding the complications of DM, 75 (16.52%) patients have experienced acute complications of DM, either diabetic ketoacidosis or hyperglycemic hyperosmolar state. Microvascular complications were seen among 84 (18.50%) patients. Retinopathy was the major (47.62%) microvascular complication followed by nephropathy (27.38%) and neuropathy (25%). Other complications were developing foot complications (21%) and non-diabetic kidney diseases (15.20%).

Overall, 193 (42.51%, 95% CI: 38.02–47.13) patients were diagnosed with CVD. Majority of them were diagnosed with hypertensive heart diseases (38.99%) and heart failure (6.83%), and the remaining experienced stroke (2.20%). The prevalence of hypertensive heart diseases was 28% (95% CI: 21–35) for younger than 40 years, 40% (95% CI: 33–46) for 40–60 years, and 49% (95% CI: 52–64) for older than 60 years patients. The magnitude of heart failure was 2.5, 8.5, and 4.4% for age <40, 40–60, and above 60 years patients, respectively. At least one risk factor for CVD was recorded among 272 (59.91%). From the total study participants, 274 (60.35%) were hypertensive. From 274 hypertensive patients, 166 (60.6%) of them were with uncontrolled blood pressure. Moreover, 124 (27.31%) were physically inactive, 28 (6.17%) were active smokers, 34 (7.49%) were alcoholics, and 83 (18.28%) were obese (Table 2).

**Table 2:** Treatment categories and magnitude of CVD risk factors among T2DM patients in government hospitals of Ethiopia, from 2013 to 2017.

**Variables**	**Category**	**Frequency**	**Percentage (%)**
Treatment	Insulin	54	11.89
	Metformin only	175	38.55
	Combined (metformin and glibenclamide)	225	49.56
Physical activities	Active	330	72.69
	Inactive	124	27.31
Cigarette smoking	Never smoked	426	93.83
	Currently smoking	28	6.17
Alcohol drinking	Never drink	420	92.51
	Currently drinking	34	7.49
Blood pressure	Normal BP	180	39.65
	Hypertensive	274	60.35
Hypertension (*n* = 274)	Controlled	108	39.4
	Uncontrolled	166	60.6
BMI	18.5–24.9 kg/m^2^	371	81.72
	Above 24.9 kg/m^2^	83	18.28
Systemic infection	No	312	68.72
	Yes	142	31.28
Blood glucose level	Good control	128	28.19
	Poor control	326	71.81
Acute complication	No	379	83.48
	Yes	75	16.52
Microvascular complications	No	370	81.50
	Yes	84	18.50

*BP, blood pressure; BMI, body mass index; CVD, cardiovascular disease; kg/m^2^, kilograms per square meter; T2DM, type 2 diabetes mellitus*.

In bivariate logistic regression, age, place of residence, physical activity, drinking alcohol, smoking, level of blood pressure, BMI, history of infection, and microvascular complications are significantly associated with CVD. However, in multivariate logistic regression, the significant association was not seen for age between 40 and 60 years, place of residence, smoking, and history of infection.

In the final multivariate logistic regression model, age older than 60, being physically inactive, drinking alcohol, HTN, BMI > 24.9 kg/m^2^, and experiencing microvascular DM complications were significantly and positively associated with CVD.

Patients with age older than 60 years were having three times [adjusted odds ratio (AOR) = 3.22; 95% CI: 1.71–6.09] higher chance of experiencing CVD as compared with those aged <40 years. The odds of developing CVD was more than two times (AOR = 2.39; 95% CI: 1.17–6.06) higher among adults who consumed alcohol compared with T2DM patients who did not drink alcohol at all. Moreover, physically inactive T2DM patients were having 45% higher odds of developing CVD (AOR =1.45; 95 CI: 1.06–2.38) when compared to the physically active patients.

On the other hand, the odds of developing CVD was 1.81 times (AOR = 1.81; 95% CI: 1.07–3.07) higher among overweight (BMI > 24.9 kg/m^2^) patients compared to patients who had normal weight (BMI 18.5–24.9 kg/m^2^). Similarly, the likelihood of acquiring CVD among hypertensive patients is more than two times (AOR = 2.41; 95% CI: 1.52–3.83) higher than those with normal blood pressure. Patients who had a history of microvascular DM complications had three times (AOR = 3.62; 95% CI: 2.01–6.53) the likelihood of developing CVD compared with T2DM patients who did not experience microvascular complications of DM (Table 3).

**Table 3:** Factors associated with cardiovascular disease among type 2 diabetes patients in government hospitals of the Harari region, Eastern Ethiopia, from 2013 to 2017 (*N* = 454).

**Variables**	**Categories**	**CVD**		**COR**	**AOR**
		**Yes**	**No**		
Baseline age in years	<40	46	109	1	1
	40–60	87	113	1.82 [1.17–2.84]	1.53 [0.93–2.54]
	Above 60	60	30	4.74 [2.71–8.28]	3.22 [1.71–6.09]^**^
Sex	Male	114	145	1	1
	Female	79	116	0.86 [0.59–1.26]	0.90 [0.71–1.83]
Place of residence	Urban	163	200	1	1
	Rural	41	87	0.58 [0.38–0.88]	0.60 [0.36–1.01]
Drinking alcohol	No	169	251	1	1
	Yes	24	10	3.51 [1.69–7.31]	2.39 [1.17–6.06]^*^
Smoking	No	174	252	1	1
	Yes	19	9	3.18 [1.46–6.92]	2.36 [0.87–6.33]
HTN	No	55	125	1	1
	Yes	138	136	2.31 [1.55–3.43]	2.41 [1.52–3.83]^**^
Physical activity	Active	119	211	1	1
	Inactive	74	50	2.56 [1.70–3.86]	1.45 [1.06–2.38]^*^
BMI	18.5–24.9 kg/m^2^	145	226	1	1
	>24.9 kg/m^2^	48	35	2.33 [1.45–3.73]	1.81 [1.07–3.07]^*^
Acute complication	No	134	236	1	
	Yes	59	25	0.61 [0.36–1.01]	0.49 [0.27–1.92]
History of infection	No	118	194	1	1
	Yes	75	67	1.96 [1.33–2.90]	1.86 [0.9–2.38]
Microvascular complication	No	134	236	1	1
	Yes	59	25	4.39 [2.66–7.24]	3.62 [2.01–6.53]^**^
Controlled blood glucose	Yes	60	68	1	1
	No	133	193	1.28 [0.88–2.01]	1.14 [0.70–1.82]

BMI, body mass index; CVD, cardiovascular disease; SD, standard deviation; AOR, adjusted odds ratio; COR, crude odds ratio; HTN, hypertension.

* p < 0.05,

** *p < 0.001*.

## Discussion

Identification of potentially modifiable associated factors of CVD is an initial step to prevent and control CVD and its outcome among T2DM patients. In this study, the overall prevalence of CVD was 42.5% (95% CI: 38%, 47%). Specific complications account for 39% hypertensive heart diseases, 7% heart failure, and 2.2% stroke. This prevalence is higher than the pooled prevalence (32%) reported in a systematic literature review of studies in the period of 2007–2017, though the specific complications are lower in the current study (37). It is also higher than those of studies conducted in Iraq (38) and Scotland (39). This discrepancy might be due to the difference in sample size, follow-up, and treatment protocol between the countries. The difference in subtypes of CVD with Malik et al. (39) and Einarson et al. (37) might be due to the wide range of age composition (younger population) in this study.

In this study, the prevalence of the CVD among T2DM is associated with the presence of older age, being physically inactive, drinking alcohol, BMI > 24.9 kg/m^2^, HTN, and presence of microvascular complications.

Patients older than 60 years have more than three times the likelihood of developing CVD than those who are younger than 40 years. The prevalence of HTN is also seen increased with age. This result is comparable to the result of studies conducted in Pakistan (40), Taiwan (41), and Sweden (42), as all of these studies revealed older age is the important predictor of a cardiovascular event in T2DM. Aging can cause changes in the heart and blood vessels that may increase a person's risk of developing CVD. Moreover, there is a high prevalence of atherosclerosis and arteriosclerosis due to the progression of diabetes in advanced age (43).

The likelihood of CVD in the female is lowered by 10% compared to male T2DM patients, though it is not statistically significant. There is heterogeneity between studies regarding the difference of risk of CVD among the male and female. Many studies indicated that the females are more likely to develop CVD (44–46). To the opposite of the former evidence from a review by Al-Salameh et al. (47), which showed that regardless of CVD being more prevalent in female in the absence of T2DM, the disparity disappears for the T2DM patients and the CVD is not barely associated with the sex difference but it is influenced by BMI above normal (overweight or obesity) and high prevalence of HTN after the age of 60–65 years in women. Hence, the reason for the non-significant association of sex and CVD in this study might be due to the higher levels of risk factors (obesity, microvascular diabetes complications, and age).

Physical inactivity is positively associated with the development of CVD among T2DM patients in this study. It is in line with the study conducted in Ethiopia among T2DM patients that indicated physical inactivity is significantly associated with the chronic complications of T2DM (48). Previous studies have consistently showed the protective effect of physical activity in diabetic patients at any level of other risk factors for CVD in diabetic patients (29, 40, 49, 50). This might be the reason for the conclusion of lifestyle modification that includes the exercise as one of the milestones of diabetes treatment and prevention of its complications (51).

Drinking alcohol is positively associated with the prevalence of CVD. The likelihood of CVD is increased by about 3-fold among alcohol consumers. Similar evidence was reported in a systematic review and meta-analysis of 20 studies (52) and individual study in southwest Ethiopia (48). Alcoholism (heavy or moderate drinking) might accelerate the development of coronary arterial diseases (CADs), as it causes systemic HTN, valvular diseases, cardiomyopathies, rhythm disturbances, and many non-cardiac problems, such as anemia, infection, and tumors (53–55). Though the risk is increased with the dose of intake and types of beverage, alcohol drinking is the risk for CVD in any individual (56).

The association between smoking and CVD is not significant in the current study. This finding is in line with a similar study conducted in Saidu Teaching Hospital, Pakistan (40). But most previous studies were reporting that smoking habit is the risk factor for the CVD, as it alters the process of controlling blood glucose level (57, 58). In INTERHEART study from 52 countries, smoking was among the reported potentially modifiable risk factors of heart diseases (59). The discrepancy might be due to the low proportion of smokers among the study population of the current study.

In the current study, there was an increment of CVD among hypertensive patients by more than two times. This study was in line with the study conducted in Sweden, as it showed that the risk of CVD is increased with the high blood pressure (31). The result from CVD research using linked bespoke studies and electronic health records (CALIBER) indicated that people with HTN had a higher lifetime risk of overall CVD and developed CVD 5 years earlier than those with normal blood pressure (30). HTN is also reported as a risk factor for CVD in published articles (31, 40–42, 60–63) through causing hypertensive heart diseases (64).

The likelihood of CVD is increased by 14% among individuals with uncontrolled blood glucose level compared to patients with controlled blood glucose. But this association was not significant statistically. To the opposite of our result, there was evidence of strong association between hyperglycemia and CVD (40, 63, 65–67). Hyperglycemia is the principal cause of microvasculopathy but also appears to play an important role in causation of macrovasculopathy (40, 68). The risk of cardiovascular complications might be increased in the long term (more than 10 years' duration); hyperglycemia as long-standing hyperglycemia induces the toxicity of endothelial cell and alters its function (11, 69, 70). The discrepancy might be due to the short period of follow-up in our study. Since the follow-up was limited to 5 years, it is rare to find the association between the uncontrolled blood glucose and CVD.

In the present study, the likelihood of developing CVD is increased by 81% among patients whose BMI is >24.9 kg/m^2^ compared to those whose BMI is between 18.5 and 24.9 kg/m^2^. A similar result was reported by a systematic review of 57 individual studies—the positive relationship between obesity and increased prevalence rates of CVD (37). This might be due to the nature of T2DM, as the majority of individuals suffering from T2DM are obese and suffer from metabolic syndrome, which could be a risk factor for the major cardiovascular events (71).

Patients who had a history of microvascular complications had an increased likelihood of developing CVD than their counterparts. Mainly recorded microvascular complications were retinopathy and nephropathy. Similarly, published studies reported the association between microvascular complication and CVD (70, 72–74). Diabetic vascular complications are a continuum and depend on each other (74). In addition to endothelial cell-dependent vascular injury mechanisms, endothelial cell-independent vascular dysfunction is leading to BK channelopathy and vascular complications in T2DM (75).

Finally, this study has a number of limitations. Since this study was a retrospective document review in a resource-limited setting, there was no recorded HbA1c, as it is not routinely available, blood cholesterol level, diet, family history, and time at CVD diagnoses for most of the patients. The coronary artery diseases, the most common types of heart disease, were not reported in the records of the patients. Moreover, the criteria used to diagnose the type of CVD are not specifically registered, and the lifetime burden of risk factors was difficult to determine.

## Conclusion

The prevalence of CVD was high and associated with advanced age, being physically inactive, drinking alcohol, BMI higher than 24.9 kg/m^2^, being hypertensive, and having microvascular complications.

## Recommendation

To reduce the risk of CVD in T2DM, helping the patients to attain a healthy lifestyle by encouraging physical activity, weight reduction, and cessation of alcohol drinking during the follow-up is the essential step. In addition to this, we recommend a multifactorial intervention aimed at achieving recommended levels of critical indicators (blood pressure, blood cholesterol, microvascular complications, and treatment at early stage).

## Data Availability Statement

All datasets generated for this study are included in the article/Supplementary Material.

## Ethics Statement

The studies involving human participants were reviewed and approved by University of Gondar College of Medical and Health Science Ethical Review Board. The patients/participants provided their written informed consent to participate in this study.

## Author Contributions

LR contributed to the proposal development, data curation, investigation, formal analysis, methodology, project administration, writing the original draft, and writing, review, and editing. AT contributed to the proposal development, investigation, methodology, writing the original draft, and writing, review, and editing. YA contributed to the proposal development, investigation, methodology, writing the original draft, and writing, review, and editing. The manuscript was also developed through the active participation of all authors. All authors read and approved the manuscript.

## Conflict of Interest

The authors declare that the research was conducted in the absence of any commercial or financial relationships that could be construed as a potential conflict of interest.
